# Disruptive mood dysregulation disorder

**DOI:** 10.1192/j.eurpsy.2021.1677

**Published:** 2021-08-13

**Authors:** P. Hervias Higueras

**Affiliations:** Psiquiatría, Hospital Universitario del Henares, Coslada, Madrid., Spain

**Keywords:** Disruptive Mood Dysregulation Disorder, bipolar disorder, Children and Adolescents

## Abstract

**Introduction:**

Faced with a possible overdiagnosis of bipolar disorder in children and adolescents, a new diagnosis has been created in the mental illness classification system. This new diagnosis is named Disruptive Mood Dysregulation Disorder.

**Objectives:**

We propose to carry out a bibliographic review on the new diagnostic category of Disruptive Mood Dysregulation Disorder.

**Methods:**

We present the clinical case of a 10-year-old boy showing severe irritability symptoms.

**Results:**

Disruptive Mood Dysregulation Disorder refers to persistent irritability and frequent episodes of extreme behavioral disturbance in children up to 12 years of age. Onset must occur before 10 years of age and the diagnosis should not be applied to children under 6 years of age. The clinical course of these patients in adolescence and adulthood tends towards unipolar depressive disorders or anxiety disorders rather than bipolar disorders.

**Conclusions:**

The new diagnosis of Disruptive Mood Dysregulation Disorder allows us to differentiate between the classic episodic presentations of mania from the non-episodic ones of severe irritability.

**Disclosure:**

No significant relationships.

